# Integrated usage of historical geospatial data and modern satellite images reveal long-term land use/cover changes in Bursa/Turkey, 1858–2020

**DOI:** 10.1038/s41598-022-11396-1

**Published:** 2022-05-31

**Authors:** Paria Ettehadi Osgouei, Elif Sertel, M. Erdem Kabadayı

**Affiliations:** 1 Institute of Science and Technology, Graduate School of Science, Engineering and Technology, ITU Ayazaga Campus, Istanbul Technical University, Sariyer, Istanbul, 34469 Turkey; 2 Geomatics Engineering Department, Civil Engineering Faculty, ITU Ayazaga Campus, Istanbul Technical University, Sariyer, Istanbul, 34469 Turkey; 3 Present Address: History Department, Koç University, Sariyer, Istanbul, 34450 Turkey; 4 History Department, Koç University, Sariyer, Istanbul, 34450 Turkey

**Keywords:** Environmental impact, Biogeography, Urban ecology

## Abstract

Land surface of the Earth has been changing as a result of human induced activities and natural processes. Accurate representation of landscape characteristics and precise determination of spatio-temporal changes provide valuable inputs for environmental models, landscape and urban planning, and historical land cover change analysis. This study aims to determine historical land use and land cover (LULC) changes using multi-modal geospatial data, which are the cadastral maps produced in 1858, monochrome aerial photographs obtained in 1955, and multi-spectral WorldView-3 satellite images of 2020. We investigated two pilot regions, Aksu and Kestel towns in Bursa/Turkey, to analyze the long-term LULC changes quantitatively and to understand the driving forces that caused the changes. We propose methods to facilitate the preparation of historical datasets for the LULC change detection and present an object-oriented joint classification scheme for multi-source datasets to accurately map the spatio-temporal changes. Our approach minimized the amount of manual digitizing required for the boundary delineation of LULC classes from historical geospatial data. Also, our quantitative analysis of LULC maps indicates diverging developments for the selected locations in the long period of 162 years. We observed rural depopulation and gradual afforestation in Aksu; whereas, agricultural land abandonment and deforestation in Kestel.

## Introduction

Global environmental change is a critical issue causing climate change, land degradation, and biodiversity loss. Land surface has been changing significantly due to natural and anthropogenic effects, causing variations in land use and land cover (LULC) characteristics of the corresponding regions. Understanding the spatio-temporal distribution, patterns, and impacts of landscape change is essential for the sustainable management of the Earth's resources^1–3^.

Agricultural land abandonment, de- and/or afforestation, and rural depopulation are acute challenges worldwide. In 2018, it was forecasted that between 2015 and 2030, about 11% of agricultural land in the European Union would be under high potential risk of abandonment^4^. Deforestation as a form of land cover change is proven to be directly linked to soil erosion and climate change^5^. Rural depopulation is becoming a central theme of research impacting both demography as well as land use^6^. A long-term perspective to understand these and other crucial forms of LULC changes is necessary. Accurate mapping of current and past land surface conditions is crucial to provide reliable geo-information for modeling the land changes, to deliver reliable inputs for different environmental models, and to develop precise decision-support systems for multi-disciplinary applications^7^.

Creating current high-resolution LULC maps became widespread with the increased availability of different satellite images and numerous image processing approaches. Many researchers have analyzed the pattern of LULC changes by using multi-temporal satellite images^7–16^. Aerial imagery and historical cadastral maps have also been used to characterize the landscape dynamics. Popelková and Mulková (2018) conducted a multi-temporal analysis of land cover change in a coalfield in today’s Czech Republic between 1836 and 2009 using cadastral maps and ortho-photos^17^. Xystrakis et al. (2017) determined post-World War II LULC change in Greece applying photo interpretation methods to aerial photographs obtained between 1948 and 2009^18^. Their results illustrated that the post-war need for agricultural production caused LULC changes within the Aetoloakarnania region. They stated that the LULC exhibits differences in vegetation densification for different periods, and agricultural abandonment was dominant between 1985 and 2007, parallel to socio-economic changes. Minta et al. (2018) used aerial photographs obtained in 1957 and 1995 and Landsat images collected in 1995 and 2014 to analyze the historical LULC changes in the central Ethiopian highlands^19^. Due to the quality limitations of their geospatial data, six LULC classes were distinguished, namely cultivated land, pastureland, forestland, woodland, settlement, and plantation land. Drummond et al. (2019) mapped the historical LULC change in northern Colorado between 1937 and 1997 of 1 m spatial resolution aerial photographs^20^. They also used historical maps and other geo-information sources to create 38 detailed LULC classes.

The generation of LULC maps from historical aerial photographs and traditional maps is challenging due to the limited spectral and radiometric resolution of aerial photographs, differences in geometry, and the definition of land classes. Cousins (2001) analyzed the land cover change in Sweden with an integrated usage of historical cadastral maps of the seventeenth and eighteenth centuries and aerial photographs from 1945 and 1981^21^. She emphasized the spatial errors inherent in old maps and the requirement of precise geometric correction to map the spatio-temporal distribution of different land cover classes accurately. She used the rubber sheet transformation method for the geometric correction of the scanned historical maps. Skaloš et al. used historical military survey maps and old ortho-photomaps to understand the long-term landscape dynamics for 250 years^22^. They determined the main problems of historical maps as geodetic inaccuracy, errors in the specification of landscape segments, and scale differences causing the loss of some spatial details. They also emphasized the challenge of finding common control points from different maps and ortho-photos due to the time difference. Seven different land cover classes used in this research are built-up areas, arable land, grassland, fruit groves, forests, water surfaces, and transport systems.

Several studies focused on the characterization of long-term LULC changes and landscape dynamics. To model long-term LULC changes, multi-modal data from various sources must be analyzed. The sources will include but are not limited to maps derived from traditional surveying methods, aerial photographs acquired from airplanes or UAVs, and satellite images^23–25^. The study by Kanianska et al. is an example of the cooperative use of historical geospatial data and very high resolution (VHR) satellite images. They analyzed the LULC changes of three rural sites in Slovakia from 1782 to 2006 using historical maps and VHR satellite data^26^. They identified a limited number of land categories from historical maps, which are agricultural land, including arable lands and permanent crops, permanent grasslands, forest, built-up areas, and others. They found significant land cover changes for the 224 years, such as permanent grassland conversion into arable and gradual afforestation of permanent grasslands and transition to the forest.

The availability of geospatial data is quite limited for the determination of land changes on a centennial time scale and these data have their specific characteristics. In general, multi-spectral VHR satellite images could be obtained after 2000 and different classification algorithms could be easily applied to these images with high performance^26–28^. High-resolution landscape characteristics of the period between the 1950s and 1970s can be generated using monochrome analog aerial photographs and Corona images from the KeyHole (KH) satellite series but these data sets have some limitations such as geometric distortions as a result of old data acquisition technology, image scanning problems and having only one spectral band. When we consider landscape mapping of early 1900s or before 1900, historical maps are the only sources that contain limitations due to the land categories available within the legend, scale problems, and exaggeration of some geographic features such as roads and water courses. For historical studies, the determination of long-term LULC change is important which requires the usage of multi-modal geospatial data from traditional maps, aerial photographs, and VHR satellite images. Dealing with various maps, aerial photographs, and satellite images challenges geometric compatibility to ensure precise locational change analysis. Also, historical LULC applications require harmonizing different geospatial aspects such as the definition of a standard projection system, finding common control points, spatial detail level, scale, and thematic content of various data sources^23,24^. Therefore, proposing methodological approaches for integrated usage of multi-source geospatial data and generating accurate LULC maps via a joint classification scheme is a challenging task since there is not much comprehensive research on this topic.

The LULC mapping has been analyzed utilizing the object-based image classification technique with high-resolution multi-spectral satellite images^27–31^. In comparison, either the available vector layers of historical maps or ortho-photos have been used, or the cadastral maps and single-band aerial photographs have been interpreted by manual vectorization^21,22^. Popelková and Mulková used the available vector layers for their spatial analysis of land cover change. In some cases, manual digitization was applied to maps or aerial photographs to form vector layers for spatial analysis^17^. Lieskovský et al. produced the first spatially explicit historical digital map of land use for the cross-border Carpathian Ecoregion, digitized from military topography maps generated between 1819 and 1980^32^. In her study of land-cover transitions in Sweden from 1945 to 1981, Cousins (2001) manually digitized black and white aerial photographs to generate grassland, arable field, water, forest, cultivated grassland, and non-management classes^21^. Skaloš et al. applied on-screen digitizing and vectorized the geo-information content of old maps and ortho-photos^22^. LULC classes used in different studies vary, but the number of classes is mostly limited due to the quality limitations of historical maps and aerial photographs. The improved and effective approach of object-based image analysis should also be utilized for generating the LULC maps from monochrome aerial photographs.

Besides the challenges of mapping the LULC from various types of geospatial datasets, the optimal methods are required to detect changes that occurred between time points of a study period. One of the most effective approaches for the optimal assessment of LULC change is the use of LULC maps from earlier dates as an auxiliary thematic layer for the analysis of the subsequent datasets. The LULC maps (e.g. classified images) of the earlier dates that consist of labeled polygons can be used to create meaningful polygons (e.g. objects) in a dataset at later dates. Gerard et al., and Yu et al. took advantage of the segmentation approach based on a land-cover map generated in an earlier date and predefining the object boundaries based on the prior parcel data to improve the detection of LULC changes^33,34^.

This study proposes a methodology to leverage the multi-modal geospatial data, including the historical datasets and VHR satellite images, to determine LULC changes for a long historical period of approximately 160 years. The first step of our process includes the geometric correction and sub-pixel spatial matching of datasets, for which we provided some suggestions. The second step is to define a joint classification schema based on the LULC classes distinguished by cadastral maps, aerial photographs, and satellite images to analyze changing landscapes. In the third step, we focused on producing accurate LULC maps from the different datasets and discussed the difficulties of creating the LULC maps from historical data sources. We suggested the iterative object-based segmentation approach to facilitate the detection of the changed areas. Finally, we assessed the trends in landscape changes and the dominant LULC change factors in our two pilot regions.

### The historical background of the study areas

**Figure 1:**
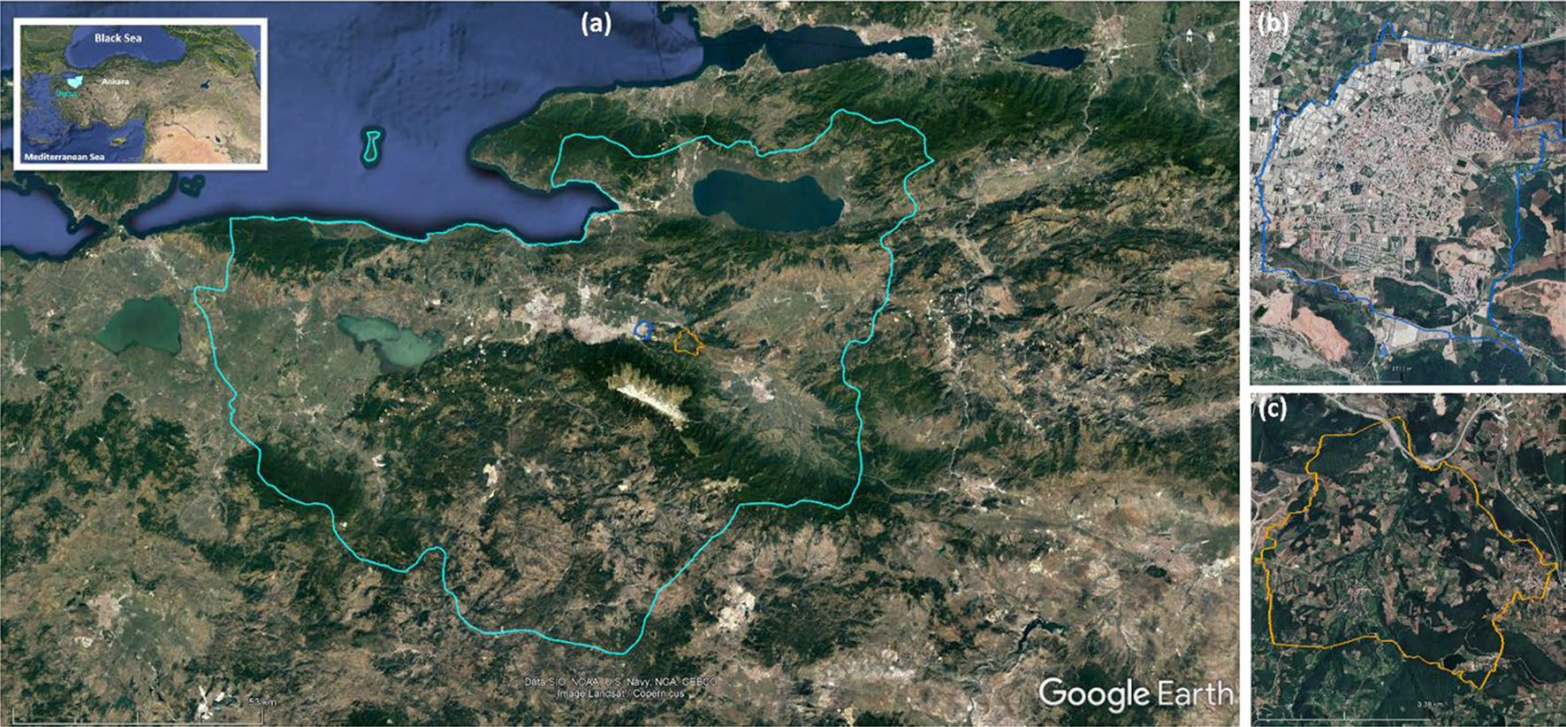
Map of the location of Bursa region in Turkey and two study sites in Bursa. (**a**) Bursa region, (**b**) Kestel, and (**c**) Aksu.

The selected towns, Aksu and Kestel, are both ancient settlements on the outskirts of the city of Bursa. Figure 1 shows the locations of Aksu and Kestel in the Bursa region. This study examines the historical LULC changes in these two towns from the 1850s until today with three time points: the 1850s, 1950s, and 2020s. Aksu was a Roman and Byzantine settlement on the Silk Road positioned as the last stop and 24 km east of Bursa. The caravanserai and the long-distance trade route determined the fate of the settlement. Aksu lived off from the caravan trade as a service providing rural settlement until the demise of this trade network in the late nineteenth century. After the end of the caravan trade, the village lost importance and turned into a self-sufficient rural settlement on the main road to Bursa until 1970. With the construction of a new route bypassing Aksu, the last episode of its long decline started. Today the village faces severe depopulation with fewer people than it had in the 1840s.

Until the late nineteenth century, Kestel remained an insignificant midway rural settlement between Aksu and Bursa, while Aksu was a small stop on a vital trade route too close to Bursa, which kept its growth potential in check. The mid-twentieth century marks the end of international migration to Kestel, the dominant source of its long-term population growth. The late twentieth century for Aksu refers to a time of stagnation and loss of logistic importance due to the shift from animal-drawn to engine-powered long-distance transport, which no longer needs to stop 24 km before or after Bursa route. In 2020, Aksu became a severely depopulated and economically deprived village administratively belonging to Kestel. Kestel, on the other hand, became an integral part of the Bursa metropolitan area and its continuous urban fabric. In this study, we used demographic data for the 1840s extracted from the Ottoman population registers available at the Turkish Presidency State Archives of the Republic of Turkey—Department of Ottoman Archives (NFS.d. collection), for 1955 publications of the Turkish Statistical Institute and for 2020 municipal sources.

## Materials and methods

### Data Used

We used cadastral maps from 1858 to reconstruct the LULC structure of Aksu and Kestel for the mid-nineteenth century. General Staff of the Ottoman Army produced these maps in 1:10,000 scale. These maps were one of the earliest attempts of creating cadastral maps in the Ottoman Empire. The images of historical maps scanned at 1270 dpi resolutions are provided by the Turkish Presidency State Archives of the Republic of Turkey – Department of Ottoman Archives (Map collection, HRT.h, 561–567). Individual tiles of cadastral maps are of a 1:2,000 scale. However, these maps are kept separated from their accompanying cadastral registers or documentation regarding their production process in the archives. There are no studies on the production of these cadastral maps, but few studies used them until now^35,36^.

The LULC structures of Aksu and Kestel for the mid-twentieth century were generated using aerial photographs from June 23, 1955, with a scale of 1:30,000. These aerial photographs were captured by the US Navy Photographic Squadron VJ-62 (established on April 10, 1952, re-designated to VAP-62 on July 1956, and disestablished on October 15, 1969). The squadron conducted mapping and special photographic projects worldwide^37^. Lastly, the VHR satellite images of WorldView-3 (WV-3) with 0.3 m of spatial resolution, collected on September 6, 2020, were used to show the up-to-date LULC patterns of Aksu and Kestel.

### Methodology

**Figure 2:**
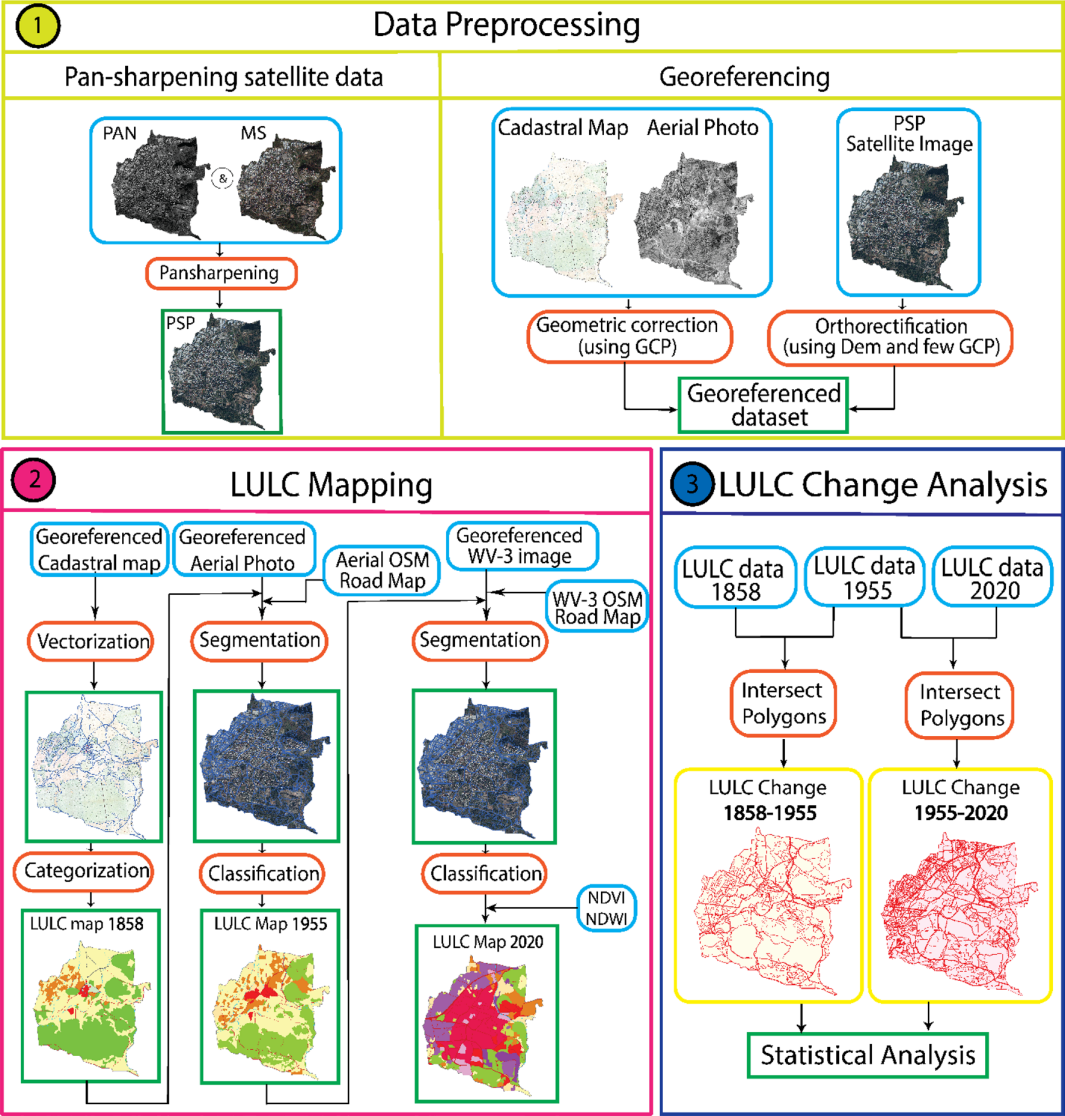
Flowchart of the processing steps for the LULC change analysis for Kestel.

Figure 2 shows the flowchart of steps followed in this study to detect the LULC changes. The workflow includes three phases: preprocessing, LULC mapping, and statistical analysis of LULC changes.

#### Data preprocessing

Orthorectification is the first important step in ensuring accurate spatial positioning among the multi-temporal and multi-source images, eliminating geometric distortions, and defining all data sets on a common projection system. To align the multi-modal geospatial datasets, we first performed the orthorectification of the satellite images and then we used the orthorectified satellite images as reference for the georeferencing of the cadastral maps and aerial photographs.

#### Satellite imagery orthorectification

We first pan-sharpened the WV-3 images by applying the PANSHARP2 algorithm^38^ to fuse the panchromatic (PAN) image of 0.3 m spatial resolution with four multispectral bands (R, G, B, and near-infrared (NIR)) of 1.2 m. We then geometrically corrected the pan-sharpened (PSP) WV-3 imageries using an ALOS Global Digital Surface Model with a horizontal resolution of approximately 30 m (ALOS World 3D – 30 m), rational polynomial coefficients (RPC) file, and additional five ground control points (GCPs) for the refinement. As a geometric model, we used the RPC model with zero-order polynomial adjustment^39^, and orthorectified images were registered to the Universal Traverse Mercator (UTM) Zone 35 N as the reference coordinate system.

#### Georeferencing of scanned cadastral maps and aerial photographs

We used orthorectified WV-3 imageries as a reference for the geometric correction of the historical cadastral maps and the aerial photographs. We selected the spline (triangulation) transformation, a rubber sheeting method, useful for local accuracy and requiring a minimum of 10 control points, as the transformation method to determine the correct map coordinate location for each cell in the historical maps and aerial photographs. The spline transformation provides superior accuracies for the geometric correction of the historical geospatial data^40,41^.

**Figure 3:**
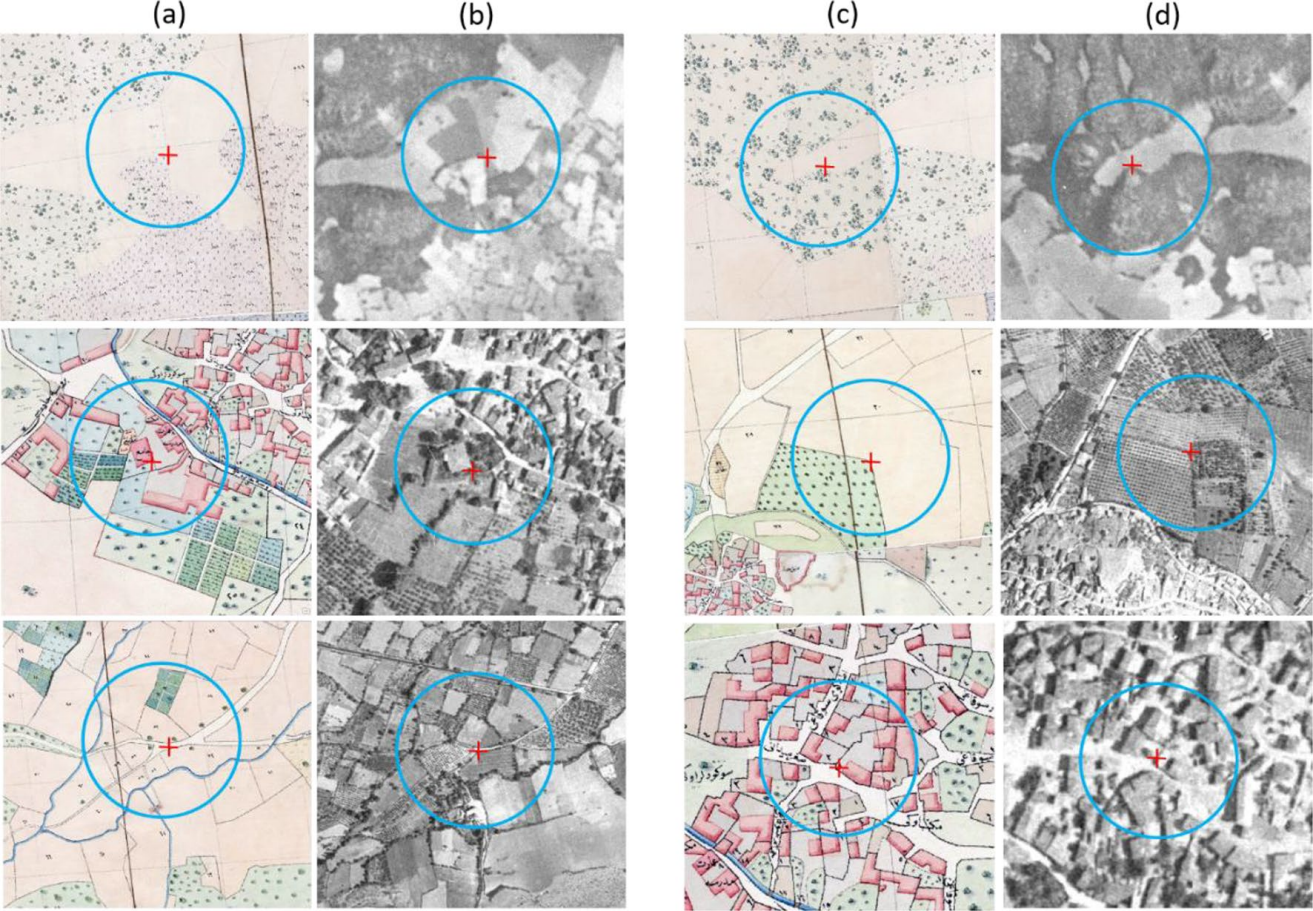
Examples of GCPs selection (red crosses in blue circles) on (**a**), (**c**) Cadastral maps and their counterparts on (**b**), (**d**) Aerial photographs.

The lack of topographic properties of planimetric features in the historical cadastral maps and the inherent distortions of the aerial photographs due to terrain and camera tilts causes difficulties in precise georeferencing of these data sets. It increases the number of required ground control points (GCPs) for optimal image rectification. Adequate and homogenously distributed GCPs, identified from cadastral maps and aerial photographs, can ensure precise spatial alignment among different geospatial data. The best locations for GCPs were border intersections of agricultural fields and roads. As for artificial objects, places of worship and schools, which are highly probable that have remained unchanged, are other optimal locations for GCPs to perform the accurate geometric correction. Figure 3 displays samples of GCPs selected from cadastral maps and aerial photographs. We obtained 2.11 m or better overall RMSE (Root Mean Square Error) values for the geometric correction of the historical maps and aerial photographs.

#### LULC classification scheme

We defined our classification scheme by analyzing the LULC classes distinguished in all three datasets (i.e., cadastral maps, aerial photographs, and WV-3 imageries). We used the classification scheme shown in Table 1. We also present codes and names of the land cover (LC) classes derived from Corine LC nomenclature^42^.

**Table 1:** Classification scheme of the study.

LC Level 1	LC Level 2	LC Level 3
1. Artificial Surfaces	1.1 Urban fabric	1.1.1 Continuous urban fabric
		1.1.2 Discontinuous urban fabric
	1.2 Industrial, commercial and transport units	1.2.1 Industrial or commercial units
		1.2.2 Road and rail networks and associated land
	1.3 Mine, dump and construction sites	
	1.4 Artificial, non-agricultural vegetated areas	
2. Agricultural areas	2.1 Arable land	
	2.2 Permanent crops	
	2.4 Heterogeneous agricultural areas	
3. Forest and semi-natural areas	3.1 Forest	
	3.2 Shrub and/or herbaceous vegetation associations	
5. Water bodies	5.1 Inland waters	

The legends provided on the historical cadastral maps of Aksu and Kestel delineate 15 LULC categories, which are: (1) buildings, (2) home gardens, (3) roads, (4) arable land, (5) gardens, (6) mulberry groves, (7) chestnut groves, (8) olive groves, (9) vegetable gardens, (10) forest, (11) courtyards, (12) vineyards, (13) arable fields, (14) cemeteries, (15) watercourses. Categorizing the land cover types of cadastral maps is limited with the classes available in the map legend. The legend of cadastral maps categorizes the forested area in one class named “forest”. Therefore, it was not possible to use third-level LC sub-categories in our classification schema for forest area which is separating forested areas into three subclasses (3.1.1, 3.1.2, and 3.1.3) according to the type of tree cover. Although some of the third-level LC sub-categories could be extracted from the cadastral map legend, it was not possible to extract all third level agricultural classes from single-band monochromatic aerial photographs. Although the spatial extent of fruit trees as a permanent crop could be determined from aerial photographs, it was not possible to classify these trees into different fruit types (e.g. 2.2.1 Vineyards, 2.2.2 Fruit trees and berry plantations, 2.2.3 Olive groves). Limitation on the number of forest classes is due to the historical cadastral map content; whereas limitation on the number of agricultural classes is mainly offset by the aerial photographs which have only one spectral band and we did not have any field survey or ancillary geographical data that could help the specific identification of fruit trees.

Our primary focus is to find out agricultural land abandonment, deforestation/afforestation, urbanization, and rural depopulation within the historical periods. Therefore, most of the second level LULC classes are sufficient for our purpose. LULC changes within the third class level such as the conversion of third level agriculture classes among each other were not aimed to analyze in this research. Our datasets allow us to use Level 3 CORINE classes for the artificial surfaces. These classes are useful to determine residential area implications of rural depopulation or urbanization, one of the focused transformation types for our analysis.

We re-organized and categorized the LULC types used in the cadastral maps, with minimum possible manipulation (only for 2.4 and 3.2 LC classes) according to the classification scheme, as shown in Table 2.

**Table 2:** Correspondence between Corine Land Cover and historical cadastral maps nomenclature.

LC class	Historical map nomenclature
1.1.2 Discontinuous urban fabric	Buildings, courtyards, home gardens,
	Courtyards
	Home gardens
	Cemeteries
1.2.2 Road and rail networks and associated land	Roads
2.1 Arable land	Non-irrigated Arable land
	Arable fields
	Vegetable gardens
2.2 Permanent crops	Gardens
	Mulberry groves
	Vineyards
	Chestnut groves
	Olive groves
2.4 Heterogeneous agricultural areas	Visual identification and categorization of land parcels principally occupied by agriculture, including both arable land and permanent crops with significant areas of natural grass and forest
3.1 Forest	Forest
3.2 Shrub and/or herbaceous vegetation associations	Visual identification and categorization of land with sparse trees
5.1 Inland waters	Watercourses

#### LULC mapping

**Figure 4:**
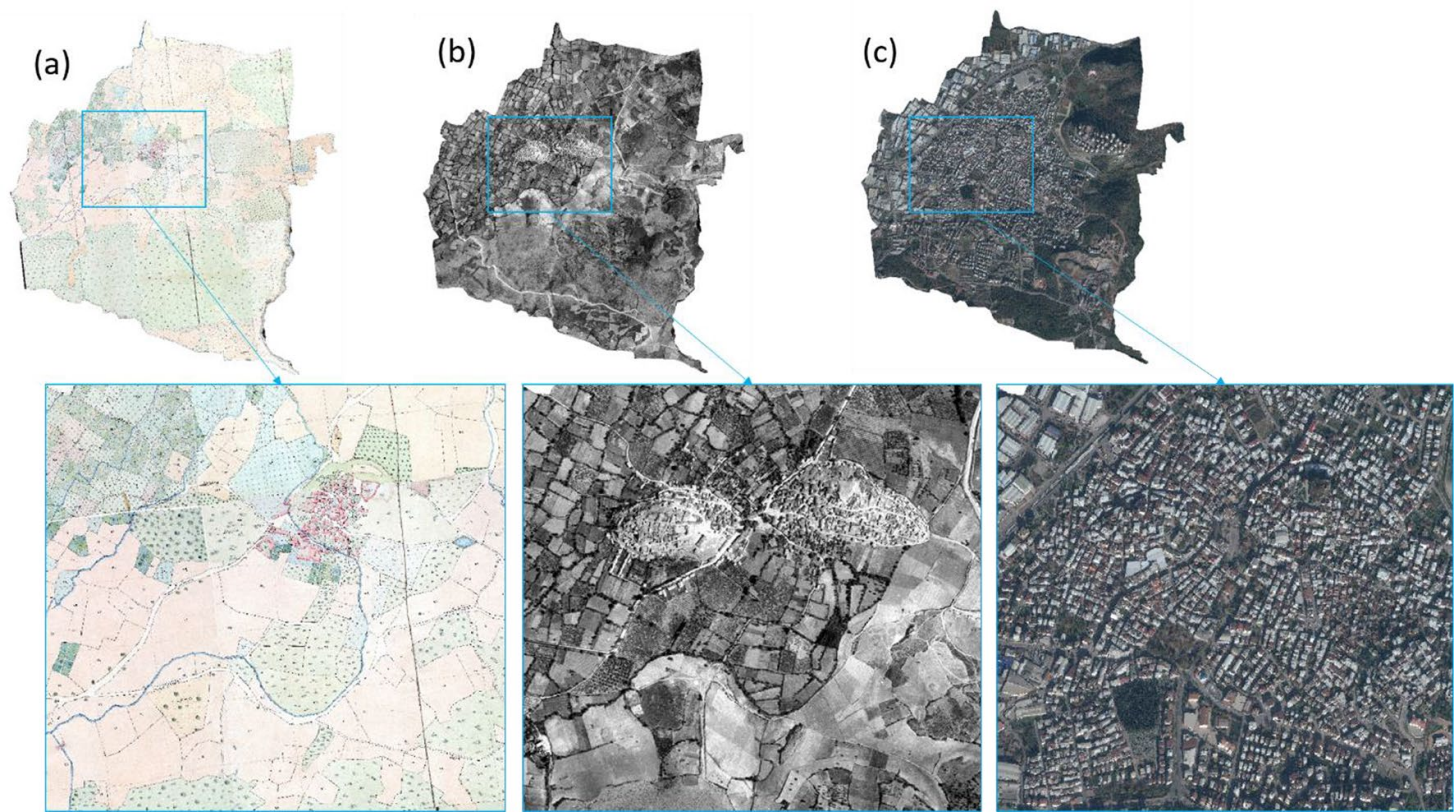
Geospatial dataset for the Kestel study region. (**a**) 1858 Cadastral map, (**b**) 1955 aerial photo, and (**c**) 2020 WV-3 satellite image (finer details shown in the inset images highlighted by Blue boxes).

**Figure 5:**
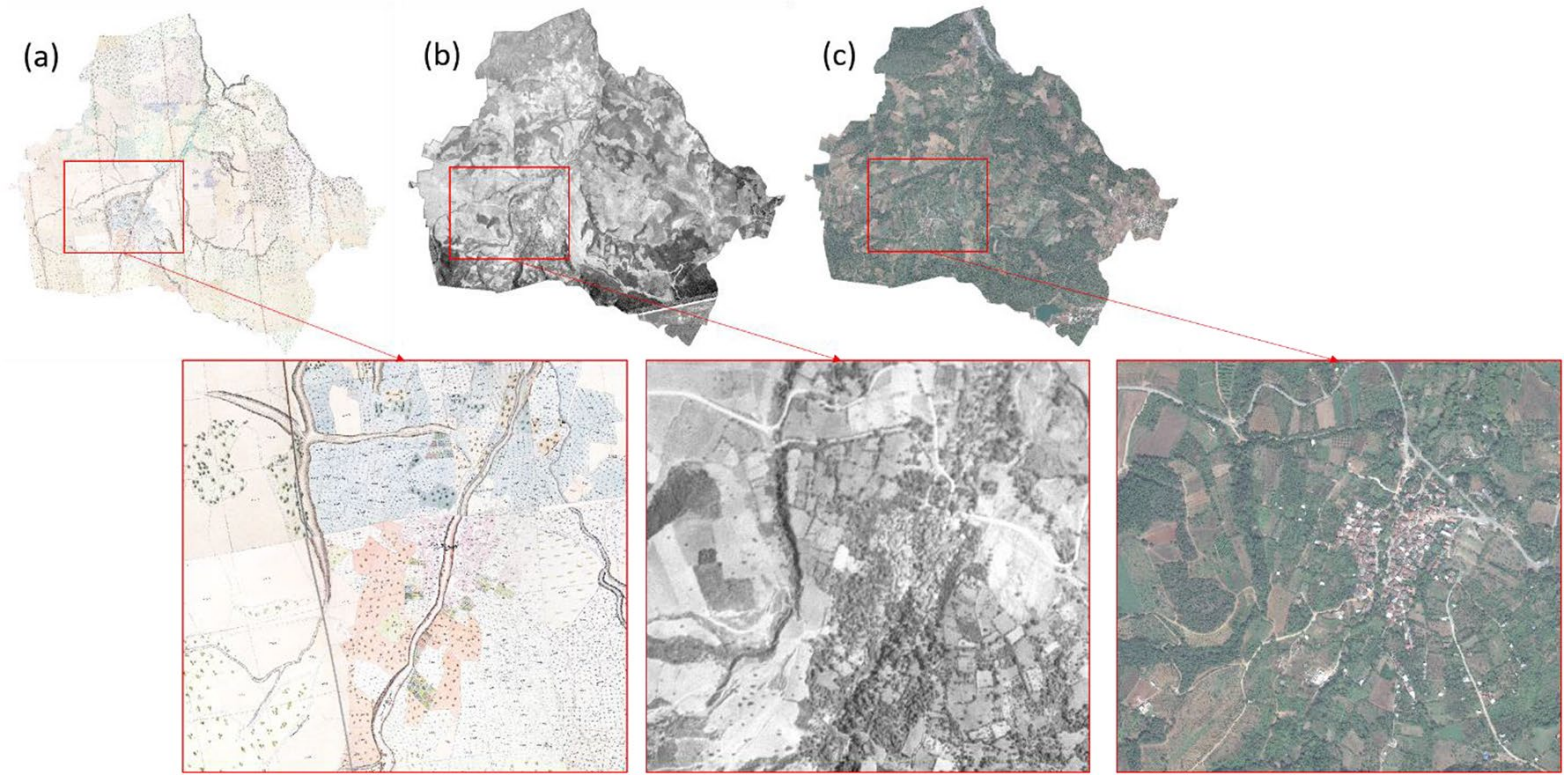
Geospatial dataset for the Aksu study region. (**a**) 1858 Cadastral map, (**b**) 1955 aerial photo, and (**c**) 2020 WV-3 satellite image (finer details shown in the inset images highlighted by red boxes).

After aligning all geospatial data, we used the georeferenced cadastral maps, aerial photographs, and satellite images for the LULC mapping. We set the spatial extent of the selected regions based on boundaries digitized from the cadastral maps of 1858. Then we detected historical LULC changes within these extents for all geospatial datasets covering 1900 ha and 830 ha of the Aksu and Kestel regions, respectively. Figures 4 and 5 show the selected extents from the historical maps, aerial photographs, and satellite images of the Kestel and Aksu sites, respectively.

#### Digitization of cadastral maps-1858 LULC maps

**Figure 6:**
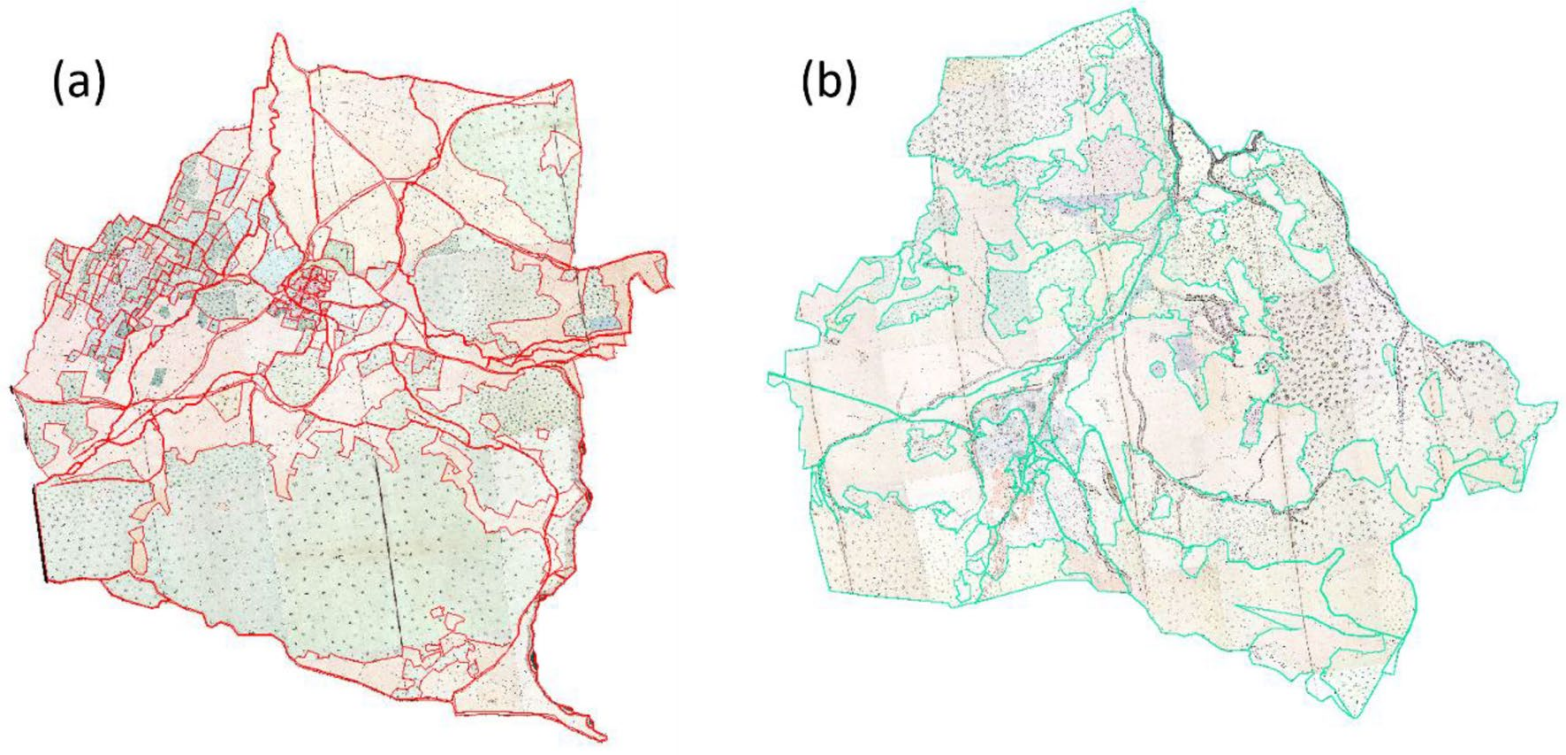
Vectorized cadastral maps of (**a**) Kestel and (**b**) Aksu with Red and green lines showing the vector boundaries.

We visually interpreted and manually digitized the geographic features on the historical maps and created vector data for each class. The road features in cadastral maps lack topological properties. They also include spatial errors possibly generated in the process of surveying and map production. Therefore, we cross-checked digitized road segments by visual inspection of the road data of the aerial photographs from 1955. We then further modified road polygons to represent accurate road widths. Afterward, we categorized vectorized features of the cadastral maps into the LULC classes defined in Table 1. Finally, we created the vectorized 1858 LULC map. Figure 6 presents the vectorized 1858 cadastral maps of Aksu and Kestel.

#### Object-based image analysis of aerial photographs-1955 LULC maps

At the second stage of LULC mapping, we performed the segmentation and classification of the aerial photographs using an object-based approach for generating the 1955 LULC map. The object-based image analysis (OBIA) approach in LULC mapping provides advantages over the traditional per-pixel techniques such as higher classification accuracy, depicting more accurate LULC change, and differentiating extra LULC classes^33,43,44^. We used the eCognition® software (Trimble Germany GmbH, Munich) to implement an object-based image analysis (OBIA). The OBIA approach contains two phases including the segmentation and classification phases that are performed to locate meaningful objects in an image and categorize the created objects, respectively.

Multiple ancillary datasets have been used to support different phases of OBIA. The Open Street Map (OSM) vector data, an open-source geospatial dataset (http://www.openstreetmap.org/), has been utilized as ancillary vector data in OBIA to improve the classification of the remotely sensed images. Sertel et al. (2018) used OSM as a thematic layer for road extraction^7^. Since there are several limitations in extracting the roads from aerial imagery, the OSM road network data could be useful. A majority of unpaved roads in single-band aerial photographs can easily be misclassified as homogeneous areas of arable lands. Precise detection of the roads from monoband aerial photographs without multi-spectral information is difficult. Therefore, we overlaid the OSM road network data with the aerial photographs to extract the revised aerial road vectors through visual interpretation and manual digitization.

We segmented the 1955 aerial photographs with the integration of 1858 LULC map produced from cadastral maps. We implemented the multi-resolution segmentation algorithm. In this segmentation method, a parameter called scale determines the size of resulting objects, and the shape and compactness parameters determine the boundaries of objects. The segmentation process of the aerial photographs was performed at multiple stages with various scale, shape, and compactness parameter values. At the initial stage, we segmented the regions according to the 1858 LULC map and we utilized large-scale parameters. The scale parameter was set to 100 and the shape parameter and the compactness were set as 0.7 and 0.3, respectively. At this stage, we focused on interpreting the objects that have not changed between 1858 and 1955. We classified the segments using the thematic layer attribute (LULC classes defined by the cadastral maps) with the highest coverage. Image objects in which the land surface has changed during 1858–1955 period were detected by visual interpretation and unclassified for further segmentation. We followed this approach to reduce the manual effort. We defined unchanged objects between 1858 and 1955 and assigned the same classes of 1858 LULC map to the objects in 1955 aerial photographs. We then segmented the remaining segments, the last time into smaller objects with the scale parameter set as 25, the shape parameter set as 0.2, and the compactness set as 0.8.

We classified the remaining unclassified objects through the development of rulesets. An object can be described by several possible features as explanatory variables which are provided by eCognition. In the classification ruleset, different features and parameters can be defined to describe and extract object classes of interest and thresholds for each feature can be defined by the trial-and-error method. We tested sets of variables for the classification of the monoband aerial photographs. Object features such as the mean value of the monoband, texture after Haralick, distance to neighbor objects, shape features (e.g., rectangular fit and asymmetry), and extent features (e.g., area and length/width) were the most useful alternatives. The classification process of the parcels of the aerial photographs with LULC change started with the classification of roads constructed between 1858 and 1955 by utilizing the aerial road map. The watercourse class was the most difficult to classify since shrubs or trees mostly covered the watercourses. These areas were misclassified as forest or agricultural land. Therefore, experts in historical map reading with local geographical information performed the detection and classification of the water course class and interpreted by the cadastral map (1858) and the google map (2020). After roads and watercourses, we classified forest and agricultural lands using the optimal thresholds for the brightness feature. We calculated the thresholds using the single band of the aerial photograph combined with the area and rectangular fit features. The heterogeneous agricultural areas class principally occupied by agriculture with significant areas of natural grass and trees within the same object are separated from the arable lands using the standard deviation of the digital number (DN) values of the aerial photographs. The texture feature helped classify the permanent crops. The brightness, shape, asymmetry, and distance to road class features were the best-performing ones for classifying the remaining artificial surfaces. The manual interpretation was performed for the classification of sub-classes of artificial surface class, including the continuous/discontinuous urban fabric, industrial, commercial, and transport units, mine, dump and construction sites, and artificial, non-agricultural vegetated areas. Since these land use classes contain one or more land cover and land use categories (e.g., artificial non-agriculture land or industrial or commercial units), finding the optimal threshold and exact feature for distinguishing the subclasses of artificial surfaces is difficult. Especially in the case of using the single-band aerial photographs, manual interpretation was required.

#### Object-based image analysis of satellite images-2020 LULC maps

We segmented WV-3 satellite images using multi-resolution segmentation algorithm and ancillary geographic data. Similar to the aerial road map, the road network of the study region in 2020, named, WV-3 road map, was extracted by overlaying the OSM road data with the WV-3 satellite image. In the segmentation process of the WV-3 image, we used the vector boundaries of the classified aerial photograph (the 1955 LULC map) and the WV-3 road map as ancillary thematic layers. We opted for the same segmentation and classification approach used for the aerial photographs for the WV-3 image.

Firstly, we segmented the satellite image into spectrally homogeneous objects using vector data of the 1955 LULC map by applying large-scale parameters. We implemented scale parameter values of 300, 200, 100, and 50 to find the optimal scale to classify objects that have not changed between 1955 and 2020. The best multi-resolution segmentation configuration was the scale of 100 and the shape and compactness parameters of 0.3 and 0.7, respectively. We classified the segments using the thematic layer attribute (LULC classes defined by the aerial maps) with the highest coverage. Segments with LULC change, e.g. the image objects in which the land surface has changed during 1955–2020 period were detected by visual interpretation and unclassified for further segmentation. As a result, we excluded the objects which were remained unchanged during 1955–2020 by assigning the prepared labels which were allocated in the previous step during the classification of 1955 aerial photographs. We then segmented the remaining objects into smaller objects to identify the changed areas in detail. At this step, the scale, shape, and compactness parameters were set as 25, 0.2, and 0.8, respectively.

Except for the additional sets of variables utilized to classify the WV-3 images, we applied the rule-set developed for the classification of the aerial photograph for the classification of the remaining objects of 2020 satellite images. The additional sets of variables include the mean of G, B, R, and NIR and two spectral indices, the Normalized Difference Water Index (NDWI), and the Normalized Difference Vegetation Index (NDVI). NDVI was calculated as the normalized difference of reflectance values in the red and NIR bands; whereas , NDWI was determined as the normalized difference of reflectance values of the green and NIR bands. Through the logical conditions, objects having specified values of NDVI and NDWI can be assigned to vegetation and water classes, respectively. The use of NDVI facilitated the delineation of terrains covered by vegetation and the NDWI improved the extraction of water bodies due to its ability to separate water and non-water objects. We separated different sub-classes of agricultural areas and forests by using optimal thresholds for NDVI which were defined by a trial and error method. Also we utilized assigning the optimal threshold to NDWI to separate water bodies from other land covers. In addition, the mean blue band layer was useful in classifying the artificial surfaces. We assessed the accuracy of each classification using error matrices (overall, user’s and producer’s accuracies, and Kappa statistics)^45,46^.

#### Estimating LULC changes and LULC conversions

After the production of LULC maps of Aksu and Kestel for 1858, 1955, and 2020, the vector data of the LULC maps were used to quantify the LULC conversions for two different periods which are 1858–1955 and 1955–2020. To compare the LULC maps of study areas between two different dates of each study period, we provided detailed “from-to” LULC change information by calculating the LULC change transition matrix computed using overlay functions in ArcGIS.

We overlaid LULC maps of 1858 and 1955 and intersected the vector boundaries of the 1858 and 1955 LULC maps to determine the conversion types of LULC classes (from which class to which class). Similarly, to quantify the LULC changes between 1955 and 2020, we overlaid the 1955 and 2020 LULC maps. Then we created transition matrices and performed statistical analysis utilizing the matrices. Finally, we discussed the main LULC change types and the driving factors of the changes in the selected study areas.

## Results

### The accuracy assessment of LULC maps

Satisfactory overall accuracies (> 85%) for the classification results are prerequisites for accurate LULC change analysis^46^. We assessed the accuracy of our classification results with a set of randomly selected reference points. We chose reference points based on two criteria: (1) We randomly selected 30 points pefo each land cover class, (2) We targeted homogenously distributed sample points within the entire mapping area. The total number of check points varies according to the number of LULC classes in LULC maps which is 300 for WV-3 and 210 for aerial imagery of the Aksu region and 360 for WV-3 and 210 for aerial imagery of the Kestel region.

We used the original aerial photographs and WV-3 images as the reference data. Table 3 presents the overall accuracy and Kappa metrics. Tables 4 and 5 show the accuracy results per-class for the aerial photographs and WV-3 images of the Aksu and Kestel regions. Of the ten LULC classes, other than arable land, permanent crop, and heterogeneous agriculture land classes in which marginal mapping confusion occurred, other LULC classes have high per-class accuracy values (> 85%).

**Table 3:** The overall accuracy and Kappa statistics.

Region	Data type	Overall accuracy (%)	Kappa(%)
Aksu	Aerial photographs	95.5	94.65
	WV-3 images	94.11	93.30
Kestel	Aerial photographs	90.74	89.16
	WV-3 images	94.72	94.10

**Table 4:** Classification accuracies for the Aksu region.

Land cover classes	WV-3 images		Aerial photographs	
	Producer's accuracy (%)	User’s accuracy %)	Producer's accuracy (%)	User’s accuracy (%)
1.1.2	100	90.48	100	96
1.2.1	100	90	–	–
1.2.2	90.63	100	96	96
1.3	100	93.33	–	–
1.4	100	100	–	–
2.1	88.57	88.57	93.02	100
2.2	97.22	100	81.82	90
2.4	86.84	94.29	93.10	90
3.1	97.06	94.29	97.44	95
5.1	100	86.67	100	96.67

**Table 5:** Classification accuracies for the Kestel region.

Land cover classes	WV-3 images		Aerial photographs	
	Producer's accuracy (%)	User’s accuracy (%)	Producer's accuracy (%)	User’s accuracy (%)
1.1.1	100	95.45	–	–
1.1.2	91.43	96.97	96.30	92.86
1.2.1	94.87	97.37	–	–
1.2.2	85.19	95.83	100.00	86.67
1.3	93.33	93.33	–	–
1.4	93.75	100	–	–
2.1	100	80	90.24	88.10
2.2	100	93.33	93.33	100
2.4	81.25	100	77.78	93.33
3.1	100.00	96.77	87.50	100
3.2	90	100	–	–
5.1	100	85	95.83	76.67

### LULC change analysis

#### LULC changes in the Aksu region in two periods: 1858–1955 and 1955–2020

**Figure 7:**
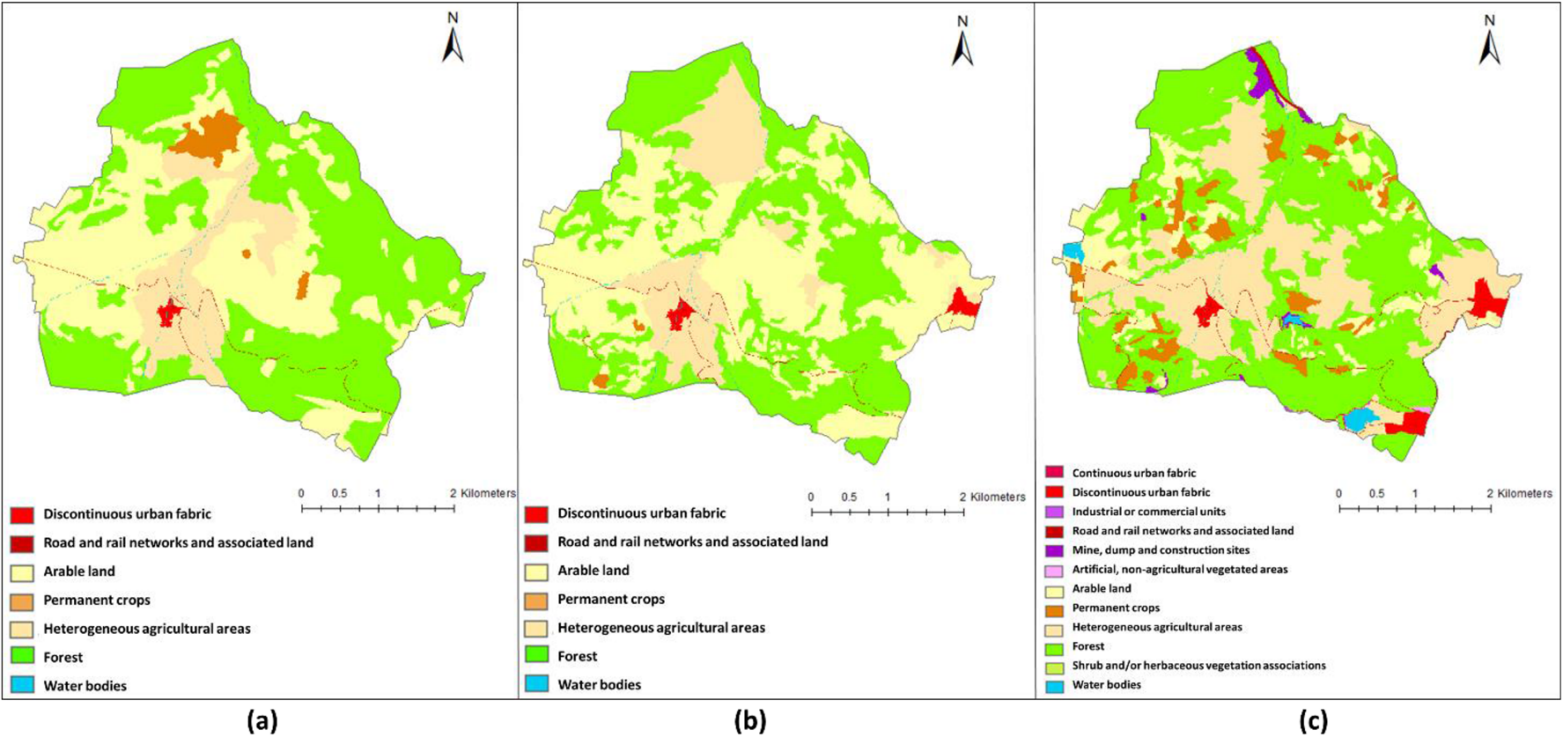
LULC maps of the Aksu region in: (**a**) 1858 (**b**) 1955 (**c**) 2020.

Figure 7 presents the LULC map of the Aksu site in 1858, 1955, and 2020, the classification results of the cadastral map (1858), aerial photograph (1955), and WV-3 imagery (2020), respectively. Tables 6 and 7 present the change matrices, showing the conversion of one LULC class to another between 1858 to 1955 and 1955–2020. In Tables 6 and 7, values in bold show the LULC classes with the change of 30% or more and identified as changed areas. Values in italics illustrate the unchanged areas for the studied periods.

**Table 6:** LULC change matrix between 1858 and 1955 in the Aksu region (areas in hectares).

LULC	1.1.2	1.2.2	2.1	2.2	2.4	3.1	5.1	Total 1858
1.1.2	*5.62*							5.63
1.2.2		*5.99*	0.03					6.09
2.1	2.36	0.17	*526.38*	1.53	**57.43**	**84.00**		671.89
2.2			1.12	*0.01*	**41.33**	0.23		42.69
2.4	1.16	0.34	**37.07**		*138.77*	**52.69**		230.06
3.1	**5.98**	0.66	**236.36**	**3.21**	**43.99**	*651.48*		941.70
5.1						0.04	*2.91*	2.99
Total 1955	15.14	7.16	800.97	4.74	281.58	788.46	2.99	1901

Significant values are in [bold, italics].

**Table 7:** LULC change matrix between 1955 and 2020 in the Aksu region (areas in hectares).

LULC	1.1.2	1.2.1	1.2.2	1.3	1.4	2.1	2.2	2.4	3.1	5.1	Total 1955
1.1.2	*14.99*		0.06					0.08			15.13
1.2.2	0.07		*6.77*			0.03		0.14	0.12		7.16
2.1	**13.88**	0.19	2.90	3.29	1.55	*194.36*	**88.95**	**322.31**	**158.95**	**14.51**	800.88
2.2							*2.60*	0.14	2.00		4.74
2.4	3.14		0.10	**9.32**		2.72	**9.04**	*194.10*	**63.04**	0.12	281.58
3.1	0.20	0.87	5.12	**7.12**	0.13	3.33	**8.16**	**11.54**	*750.03*	1.81	788.31
5.1								0.10	0.24	*2.63*	2.99
Total 2020	32.28	1.07	14.96	19.73	1.69	200.44	108.75	528.42	974.37	19.09	1901

Significant values are in [bold, italics].

The LULC maps (Fig. 7) show that the region is mostly covered by forest (LC class 3.1) and agricultural areas (LC classes 2.1, 2.2, and 2.4), and LULC change was relatively minor in the entire period. The agricultural land (944.63 ha) in 1858 was dominated by arable land (2.1) (671.89 ha), followed by heterogeneous agricultural area (2.4) (230.05 ha) and permanent crops (2.2) (42.68 ha). In 1955, the agricultural areas increased to 1087.30 ha with the conversion of 236.36 ha forest area to agricultural area (Table 6) which was also dominated by arable land (800.97 ha).

In 2020, the agricultural area (LC class 2) (838 ha) was dominated by heterogeneous agricultural areas (2.4) (529 ha). Also, there has been a 24% increase in the forest area (3.1) between 1955–2020 with the conversion of 159 ha of arable lands and 64 ha of heterogeneous agricultural area (2.4) to the forest area. Artificial surfaces (LC class 1) covered only 12 ha, 22 ha, and 70 ha in 1858, 1955, and 2020, respectively, which indicates a low anthropogenic impact on the study area within 163 years (Fig. 7, Tables 6 and 7).

#### Driving forces of LULC changes in the Aksu Region

The almost stagnant population of Aksu increased slightly from 472 inhabitants in the 1840s to 658 inhabitants in 1955. Only a 40% increase in more than a century for population, which normally grows exponentially, is a sign of rural depopulation in the long-term. Still, the minute population growth is in accord with the conversion of forest lands to agricultural areas between 1858 to 1955 due to the increasing human activities and, specifically, agriculture practices. After the construction of the new main Bursa road in 1972, the older road passing through Aksu village lost its importance, and economic activities in the village diminished significantly. The population of Aksu decreased to just 362 inhabitants in 2020. The drastic depopulation in the second half of the twentieth century explains the most significant LULC change between 1955 and 2020 in the Aksu region, which is the transformation of arable lands (2.1) to heterogeneous agricultural areas (2.4) and forest (3.1). The arable lands were transformed to lands covered by a mixture of shrubs and annual grasses and plants due to gradual forestation in the region after agricultural land abandonment. In sum, rural depopulation accompanied by agricultural land abandonment set the LULC dynamics in the long period under consideration for the Aksu region.

#### LULC change in the Kestel Region in two periods: 1858–1955 and 1955–2020

**Figure 8:**
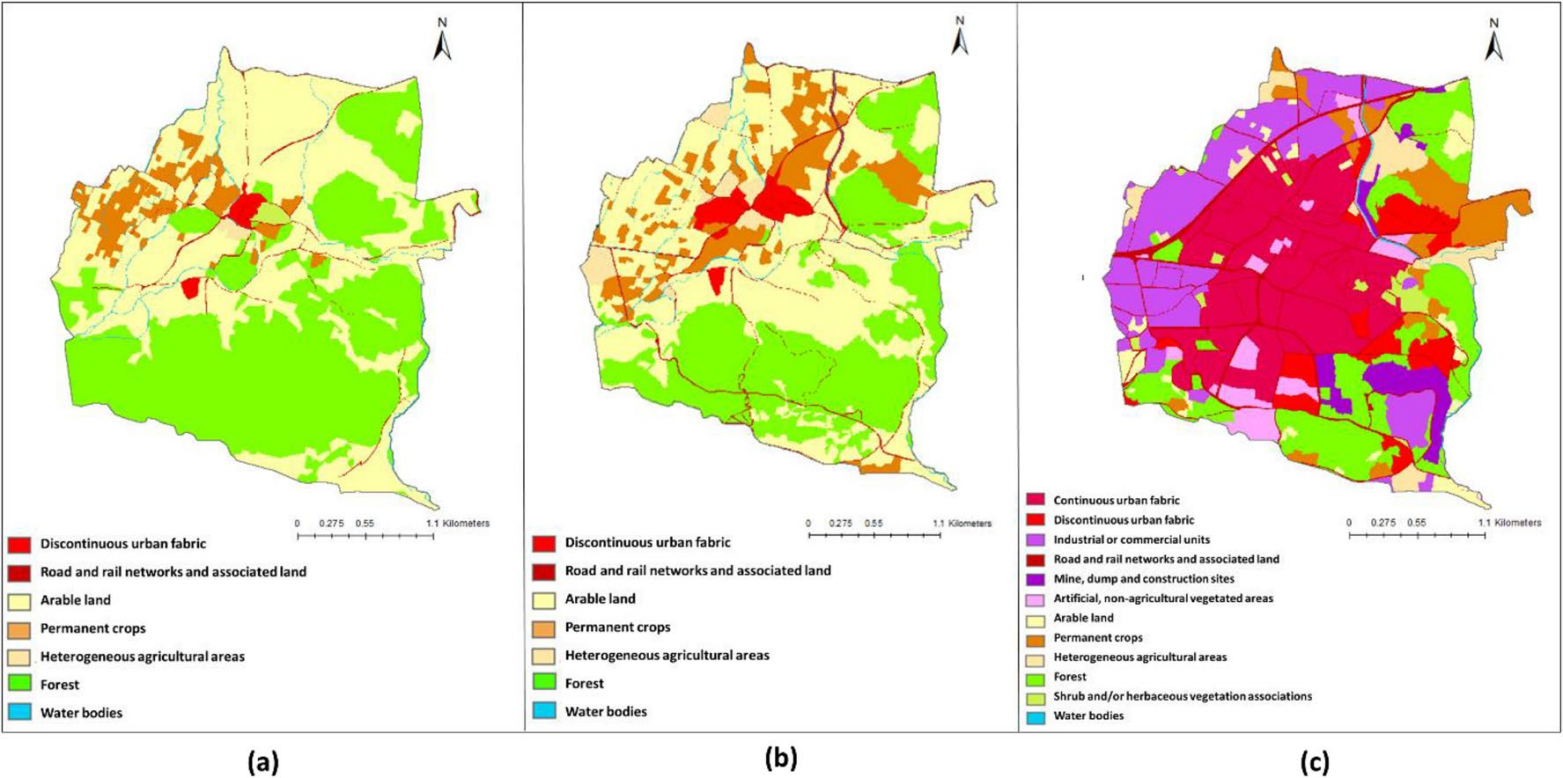
LULC maps of the Kestel region in: (**a**) 1858, (**b**) 1955, (**c**) 2020.

Figure 8 presents the LULC map of Kestel in 1858, 1955, and 2020, based on the classification of the cadastral map (1858), aerial photograph (1955), and WV-3 image (2020). Tables 8 and 9 present the change matrices for two periods (1858–1955, 1955–2020). In Tables 8 and 9, cells with top 30% values are highlighted in bold, and cells in italics show the total area of LULC class remained unchanged during each period. In 1858, the agricultural areas (LC class 2) and forest and semi-natural areas (LC class 3), were dominant in the LULC structure of the Kestel, constituting approximately 49% and 48% of the study area, respectively. The remaining 3% of the study area is covered by discontinuous urban fabric (LC class 1.1.1), road and rail networks, and associated land (LC class 1.2.2), and watercourses (LC class 5.1). The most significant LULC changes between 1858 and 1955 were related to forest and semi-natural areas in which forest land decreased from 394 ha in 1858 to 272 ha in 1955. During 98 years between 1858 and 1955, 260 ha out of 390 ha of forest lands in 1858 remained unchanged, while 116.34 ha transformed to agricultural land and 16.7 to discontinuous urban fabric (LC class 11.2) and roads (LC class 1.2.2) (Table 8). Discontinuous urban fabric class increased from 0.85% in 1858 to 3% in 1955, which mostly captured the forest class (LC class 3.1) (-7.27 ha) and arable land class (LC class 2.1) (-2.6 ha).

**Table 8:** LULC change matrix between 1858 and 1955 in the Kestel region (areas in hectares).

LULC	1.1.2	1.2.2	2.1	2.2	2.4	3.1	5.1	Total 1858
1.1.2	*6.79*	0.28						7.07
1.2.2	0.39	*8.59*	0.39	0.09	0.12	0.03	0.01	9.62
2.1	2.57	**6.76**	*249.90*	**74.47**	**13.15**	**11.55**	2.48	360.89
2.2	1.64	0.21	**31.22**	*8.24*	3.60		0.15	45.06
2.4	0.05	0.05		0.30	*2.81*			3.22
3.1	**7.27**	**5.54**	**100.46**	**13.50**	2.20	*260.30*	0.76	390.21
3.2	3.48	0.42						3.90
5.1	2.00	0.33	1.64	0.28	0.17	0.34	*4.24*	9.01
Total 1955	24.19	22.18	383.80	96.90	22.04	272.22	7.65	829

Significant values are in [bold, italics].

**Table 9:** LULC change matrix between 1955 and 2020 in the Kestel region (areas in hectares).

LULC	1.1.1	1.1.2	1.2.1	1.2.2	1.3	1.4	2.1	2.2	2.4	3.1	3.2	5.1	Total 1955
1.1.2	**20.98**			0.37		0.71							22.06
1.2.2	3.24	0.33	0.98	*11.54*	0.82	1.9	0.2	0.91	0.3	0.98	0.22	0.58	22
2.1	**114**	**22.78**	**87.31**	**23**	6.29	6.19	*32.78*	**32.01**	**22.07**	**22.23**	9.15	0.23	378.04
2.2	**27.88**	1.17	**28.76**	5.8	0.64	4.55	0.8	*18.82*	4.67	0.72	0.45		94.26
2.4	9.95		10.88	0.58			0.66				0.02		22.09
3.1	**39.12**	**28.92**	**16.8**	**12.78**	**25.39**	**23.37**	2.4	7.18	11.49	*113.7*	1.38	0.39	282.92
5.1	1.4	0.02	2.29	0.75	0.1	0.28	0.08	0.14	0.55	0.08	0.12	*1.79*	7.6
Total 2020	216.57	53.22	147.02	54.82	33.24	37	36.92	59.06	39.08	137.71	11.34	2.99	829

Significant values are in [bold, italics].

Shrub and/or herbaceous vegetation associations class (LC class 3.2) including natural grassland, herbaceous vegetation, and scattered trees located on the east of the urbanized region of Kestel in 1858 with a total area of 3.48 ha, which then converted to the discontinuous urban area during the period of 1858–1955. Also, the forest land parcel covering the 7.30 ha at the west of the urbanized region of Kestel in 1858 was converted to a discontinuous urban area in the same period. In addition, the road constructions occupied approximately 6 ha of forest and semi-natural areas and close to 7 ha of agricultural areas between 1858 and 1955.

Visual interpretation of LULC maps of the Kestel region in 1955 and 2020 shows that the initial spatial structure of this town was changed fundamentally during the years between 1955 and 2020 (Fig. 8). The study area in 1955 was covered mainly by agricultural areas and forest, whereas the built-up area, majorly aggregated in the center of the town made up a minuscule portion (5.3%) of the study area. In 2020 land use changed drastically. The built-up area, including urban structures and industrial constructions, expanded significantly, and a large amount of farmland and forest was replaced with the urban fabric. The most significant land-use change in Kestel during 65 years between 1955 and 2020 is a significant decrease in agricultural areas (from 59.6% in 1955 to 16.3% in 2020) and forest and semi-natural areas (from 34.1% in 1955 to 17.9% in 2020). Only 32.7 ha (8.6%) of arable land class in 1955 with a total area of 378 ha have remained unchanged in 65 years between 1955 and 2020. 36.1% of the arable land class was transformed to the urban fabric (LC class 1.1), 29.1% to industrial, commercial, and transport units (LC class 1.2), 1.66% to mine, dump, and construction sites (LC class 1.3), 1.63% to artificial, non-agricultural vegetated areas (LC class 1.4) (Table 8). The percentage of artificial surfaces (LC class 1) in the Kestel region was 5.3% with a total area of 44 ha in 1955, and it increased in 65 years ten-fold to almost 497.8 ha. As a result, the percentage of artificial surfaces was 65.3% with a total area of 541.8 ha in 2020 (Fig. 8, Table 9). The Kestel study area had changed substantially by the industrial activities during 1955 and 2020 in which the 126.9 ha of agricultural area and 16.8 ha of forest land were transformed to the lands that are currently under industrial or commercial use.

#### Driving forces of LULC changes at the Kestel Region

In addition to biological reproduction, Kestel gained population due to migration in around a hundred years between our first two observation years. Bursa region, in general including towns such as Kestel, were arrival points for waves of migration from the Balkans both in the late 19th as well as in the early twentieth century^47^. Its population grew from 228 inhabitants in the 1840s to 2359 inhabitants in 1955. This population growth caused an increase in the agricultural areas between 1858 and 1955 (from 409 to 503 ha), which can be explained by increased agricultural activity and consequent deforestation (− 116.34 ha of forest class) in the region. Due to the lack of systematic mechanization in agricultural production in Turkey and the Bursa region until the 1950s, we can safely assume that increased agricultural activity resulted in an extension of agricultural land instead of an intensification of production based on technological change. Migration could also explain a marginal increase of discontinuous urban fabric in the region. The discontinuous urban area in 1858 grew to the east into the land, which was covered with a natural grassland, and to the west into the forest land. The more drastic LULC changes in the second half of the twentieth century in the Kestel region are related to the further increased human activity. The last wave of immigration from Bulgaria in 1989 caused another jump in the population of Kestel and accelerated urban growth in the 1990s. Kestel town experienced the most recent LULC changes after it became one of the seven districts within the borders of the Bursa province in 2004, resulting in final population growth (from 2359 inhabitants in 1955 to 70,865 inhabitants in 2020). The proximity to the industrialized city of Bursa, economic growth, and ever-increasing employment opportunities due to the industrial potential of Kestel further accelerated the rate of LULC change in this region. Today the Kestel town borders the Bursa city to the west, where most of its industrial sites are located. It is plausible that Kestel will be an organic part of the urban amalgamation of the city of Bursa in the near future. In sum, the LULC changes of Kestel were driven by accelerated urbanization sustained by long-term immigration.

## Discussion

### LULC dynamic of Aksu and Kestel during 1858–2020

Historical cadastral maps and aerial photographs in companion with VHR satellite imageries allowed us to track long-term LULC changes from 1858 to 2020 in two study areas in Bursa/Turkey, namely Aksu and Kestel sites. While we digitized the scanned cadastral maps, we classified the aerial photographs and satellite images using the OBIA approach and generated LULC maps for 1858, 1955, and 2020. The overall accuracy values are higher than 90% (Table 3). For most of the LULC classes UA and PA values are higher than 90% (Tables 4 and 5). We obtained very high accuracy values for artificial surface, water and forest classes for 2020 LULC maps with the important contribution of 30 cm spatial resolution of WV-3 images. According to the statistical analysis of the LULC changes, Kestel has experienced significant land changes compared to Aksu between 1858 and 2020. The primary trend observed during the 163 years at Kestel is the significant agricultural area and forest conversion into urban land and industrial sites, where forest and semi-natural areas decreased from around 48% in 1858 to approximately 18% in 2020. The agricultural areas decreased from around 49% in 1858 to roughly 16% in 2020. In the meantime, the LULC of Aksu was changed only slightly in the same period. A gradual forestation and arable land conversion to heterogeneous agricultural areas were the main characteristics, where heterogeneous agricultural areas increased from around 12% in 1858 to roughly 28% in 2020. The artificial surfaces increased significantly from only 2% in 1858 to approximately 66% in 2020 in Kestel. In contrast, they rose only marginally from 0.62% in 1858 to 3.66% in 2020 in Aksu.

The socio-economic, demographic, and technological factors influencing the LULC changes can be analyzed in two periods between 1858 and 2020: (1) 1858–1955 and (2) 1955–2020. The dynamics of agricultural and forest land changes due to the dependence of an economic livelihood on farming impacted the LULC changes between 1858 and 1955. In the first period, the slight increase in population due to migrations and following human activities led to the conversion of forest lands to agricultural areas. Therefore, the most apparent change in the first period at both sites was an expansion of agrarian land based on extensive agriculture. In contrast, in the second period, 1955–2020, the socio-economic and technological factors became more critical, and migration, urban expansions, and industrial development mainly characterized the period. In this period, Aksu and Kestel followed radically diverging paths. The population growth due to immigration and natural increase and industrial area developments due to the proximity to the Bursa city center intensified the LULC changes in Kestel. On the contrary, lack of immigration and loss of logistical importance led to drastic rural depopulation, widespread agricultural land abandonment, and gradual afforestation in Aksu.

### Iterative classification approach

We used the object-based classification method to segment the remotely sensed images, including the single-band aerial photographs and multi-spectral satellite imageries, into discrete objects and then applied decision trees for the identification and classification of these objects. In our proposed approach, we benefitted from the LULC outputs of former date for the consecutive segmentations. We utilized the vector boundaries of polygons in the classified cadastral maps as ancillary data for the segmentation and of the aerial photographs which improved the overall accuracy. We reduced the manual effort by using the labels 1858 LULC maps to assign the class to objects in aerial photographs which have not changed during 1858 and 1955.

Consistently, we used the classification vectors of aerial photographs to segment the satellite imageries into meaningful objects for LULC detection and utilize the classified 1955 aerial photo to label the objects in 2020 satellite images that have not changed during 1955 and 2020. Additionally, we used the OSM road network to improve the classification of the road class. Overall, integration of the digitized cadastral maps, former date classification results, and open-source geo-information into the object-based classification of aerial photographs and satellite images maps minimized the manual effort for the visual interpretation of aerial photographs and improved the classification accuracy for both data sets.

### Drawbacks of historical datasets

There are some limitations in using the historical cadastral maps and aerial photographs for LULC change analysis. Traditional cadastral maps do not contain topographic properties of planimetric features; therefore, there is no information on relief causing difficulties in georeferencing. Possible deterioration of image quality caused by scanning the original hardcopy of aerial photographs and the inherent distortions caused by terrain and camera tilts preclude the precise rectification and classification of the aerial photographs. Moreover, classes used for long term land change analysis are highly dependent on the legend of the historical traditional map and limitations of aerial photographs specifically on the spectral domain. Although we can extract higher details for artificial surface classes, we cannot able to extract detail agricultural and forest classes due to these limitations.

### Limitations of the approach for LULC change detection

Although the literature mainly utilizes object-based image analysis methods to classify VHR multi-spectral satellite images and manual methods for aerial photographs, our results showed that a hybrid approach of manual and object-based techniques is very useful for the classification of single-band aerial photographs. Our method can be time-efficient and provide more accurate results. Even though some manual interpretations are yet required, object-based segmentation and classifications significantly facilitate the LULC change detection in this study. Studies such as Yu et al. (2016) suggest utilizing various methodologies like the change vector analysis (CVA) to identify areas with changes automatically but this approach still requires multi-spectral images of similar sources for different periods^33^.

## Conclusion

We evaluated the integration of historical geospatial data and recent VHR satellite images to determine the historical LULC changes between 1858 and 2020. Our results showed that a hybrid approach of manual and object-based techniques is very useful for the classification of single-band aerial photographs. Iterative use of the classified geospatial data of an earlier date for segmentation and classification of the data in a subsequent date facilitates both the generation of LULC maps and the detection of LULC changes.

Despite some limitation of the aerial photographs and historical cadastral maps including inherent geometric distortions, lack of topographic properties, and deterioration of image quality caused by scanning the original hardcopy; these historical geospatial data provide valuable information about the historical spatial distribution of LULC classes to understand the past landscape conditions and how these areas have changed due to different factors. LULC classes that will be evaluated need to be extracted from all data sets. Therefore, they are limited with the characteristics of traditional maps and aerial photographs.

Multi-temporal LULC maps could be used to predict future landscape conditions and analyze the changes under similar driving forces. Different researchers could apply our proposed approach to generate highly accurate LULC maps of other regions from similar multi-modal geospatial data. In future studies, optimal automatic LULC change detection methods using multi-source geospatial data including single-band aerial photographs and multi-spectral satellite images will be further studied to minimize the manual interpretation. Finally, our proposed approach could be implemented to create reliable labeled reference datasets for deep learning models to automatize historical LULC mapping studies.

## Data Availability

Original geospatial data including cadastral maps, aerial photographs and WV-3 satellite images are not publicly available due to copyright rules of data providers but are available from the corresponding author on reasonable request. Newly generated LULC data are included in this published article as high resolution figures. Original versions of these LULC maps are available from the corresponding author on reasonable request.
